# CSF-1R as an inhibitor of apoptosis and promoter of proliferation, migration and invasion of canine mammary cancer cells

**DOI:** 10.1186/1746-6148-9-65

**Published:** 2013-04-05

**Authors:** Magdalena Król, Kinga Majchrzak, Joanna Mucha, Agata Homa, Małgorzata Bulkowska, Arleta Jakubowska, Malwina Karwicka, Karol M Pawłowski, Tomasz Motyl

**Affiliations:** 1 Department of Physiological Sciences, Faculty of Veterinary Medicine, Warsaw University of Life Sciences - WULS, Nowoursynowska 159, Warsaw, 02-776, Poland; 2 Department of Animal Environment Biology, Faculty of Animal Sciences, Warsaw University of Life Sciences - WULS, Ciszewskiego 8, Warsaw, 02-786, Poland; 3 Department of Large Animal Diseases with Clinic, Faculty of Veterinary Medicine, Warsaw University of Life Sciences – WULS, Nowoursynowska 100, Warsaw, 02-797, Poland

**Keywords:** Canine mammary carcinoma, CSF-1R, Tumor-associated macrophages, Tumor microenvironment

## Abstract

**Background:**

Tumor-associated macrophages (TAMs) have high impact on the cancer development because they can facilitate matrix invasion, angiogenesis, and tumor cell motility. It gives cancer cells the capacity to invade normal tissues and metastasize. The signaling of colony-stimulating factor-1 receptor (CSF-1R) which is an important regulator of proliferation and differentiation of monocytes and macrophages regulates most of the tissue macrophages. However, CSF-1R is expressed also in breast epithelial tissue during some physiological stages i.g.: pregnancy and lactation. Its expression has been also detected in various cancers. Our previous study has showed the expression of CSF-1R in all examined canine mammary tumors. Moreover, it strongly correlated with grade of malignancy and ability to metastasis. This study was therefore designed to characterize the role of CSF-1R in canine mammary cancer cells proliferation, apoptosis, migration, and invasion. As far as we know, the study presented hereby is a pioneering experiment in this field of veterinary medicine.

**Results:**

We showed that *csf-1r* silencing significantly increased apoptosis (Annexin V test), decreased proliferation (measured as Ki67 expression) and decreased migration (“wound healing” assay) of canine mammary cancer cells. Treatment of these cells with CSF-1 caused opposite effect. Moreover, *csf-1r* knock-down changed growth characteristics of highly invasive cell lines on Matrigel matrix, and significantly decreased the ability of these cells to invade matrix. CSF-1 treatment increased invasion of cancer cells.

**Conclusion:**

The evidence of the expression and functional role of the CSF-1R in canine mammary cancer cells indicate that CSF-1R targeting may be a good therapeutic approach.

## Background

Cancer cell produces various cytokines and chemokines that attract leukocytes in a similar manner as a site of tissue injury, where monocytes migrate guided by chemotactic factors [1,2]. Tumor-associated macrophages (TAMs) have high impact on cancer development because they are educated by the tumor microenvironment to facilitate matrix invasion, angiogenesis, and tumor cell motility [3]. It gives cancer cells the capacity to invade normal tissues and metastasize [3,4]. The signaling of colony-stimulating factor-1 receptor (CSF-1R) which is an important regulator of proliferation and differentiation of monocytes and macrophages [5] regulates most of the tissue macrophages. However, CSF-1R is expressed also in breast epithelial tissue during some physiological stages e.g.: pregnancy and lactation. Its expression is also detected in some cancers [6] but its role in cancer cell biology is not fully recognized. Data reported in the literature indicated that the oncogenic potential of CSF-1R is due to co-expression of this receptor and its ligand CSF-1 (colony stimulating factor-1) in epithelial cancer cells, or mutations activating CSF-1R independently of ligand [7]. Activation of CSF-1R by its ligand is likely to occur in an autocrine manner in tumor cells in which CSF-1R and CSF-1 are co-expressed, or in paracrine manner, when CSF-1R is stimulated by CSF-1 released by fibroblasts [6]. However, our recent studies have shown that CSF-1R in cancer cells can be stimulated by colony stimulating factor secreted by tumor-associated macrophages [8]. Interestingly, we showed that their co-culture leads to up-regulation of CSF in macrophages and up-regulation of CSFs and CSF-1R in cancer cells. These results are in accordance with the observations that CSF-1R is important for cancer metastasis but only in the presence of macrophages [9]. Moreover, our previous study [10] has shown that the expression of CSF-1R in neoplastic epithelial cells of canine mammary gland strongly correlates with grade of malignancy and ability to metastasis. This study was therefore designed to characterize the role of CSF-1R in canine mammary cancer cells proliferation, apoptosis, migration, and invasion. As far as we know, the study presented hereby is a pioneering experiment in this field in veterinary medicine.

## Methods

### Cell lines

The cell lines used for the study have previously been used in other published research [8,11-14]. Two canine mammary adenocarcinoma cell lines (CMT-W1, CMT-W2), anaplastic cancer cell line (P114), simple carcinoma cell line (CMT-U27) and spindle-cell mammary tumor cell line (CMT-U309) were examined. CMT-W1 and CMT-W2 cell lines were kindly donated by Prof. Dr. Maciej Ugorski and Dr. Joanna Polanska from Wroclaw University (Poland), CMT-U27 and CMT-U309 cell lines were kindly donated by Dr. Eva Hellmen from Swedish Agricultural University (Sweden) and P114 cell line was kindly donated by Dr. Gerard Rutteman from Utrecht University (The Netherlands).

Cells were cultured under optimal conditions: in RPMI-1640 medium enriched with 10% (v/v) heat-inactivated fetal bovine serum (FBS), penicillin-streptomycin (50 iU mL–1), and fungizone (2.5 mg mL–1) (reagents obtained from Sigma Aldrich, USA), in an atmosphere of 5% CO2 and 95% humidified air at 37°C. At the time of experiments, the culture conditions were changed as follows: the cells were grown in RPMI-1640 medium without FBS for 24 hrs, and then the medium was replaced with normal growth medium supplemented with CSF-1 at the concentrations of 25 ng/ml, 50 ng/ml, or 100 ng/ml [6]. Because our previous study [8] showed that adding of macrophages (secreting CSF-1) to cancer cells culture (grown in RPMI-1640 medium enriched with 10% FBS) increased the expression of CSF-1R in cancer cells, and caused significant changes in their behavior, we conducted the presently described experiments in a similar manner (cancer cells grown in full medium) using CSF-1 instead of macrophages. This experimental model enabled to answer the question whether macrophages exert their influence on cancer cells via CSF-1/CSF-1R.

### siRNA transfection

The siRNA transfection procedure used in canine mammary cancer cells was described in details in our previously published study [14]. The cell density, transfection reagent toxicity and transfection efficacy were optimized according to the procedure described in our previous manuscript: [14]. The canine (*Canis lupus familiaris*) *csf-1r* sequence was obtained from Gene Bank with accession number [XM_546306.3]. The siRNA duplexes were designed by http://www.sigmaaldrich.com/life-science/custom-oligos/sirna-oligos/sirna-design-service.html. The results were confirmed using two independent algorithms: Dharmacon (OligoWalk) and Ambion and at last two duplexes were chosen for further experiments (obtained from Sigma Aldrich) (1^st^ duplex sequences, are as follow: GUGAGAAGGUCGAUCUCCAdTdT and UGGAGAUCGACCUUCUCACdTdT; 2^nd^ duplex sequences, are as follow: CACAAUCCCUCAACAAUCUdTdT and AGAUUGUUGAGGGAUUGUGdTdT). For *csf-1r* silencing the mixture of both duplexes was used (30 pmol + 30 pmol).

All the experiments with transfected cells were conducted 48 hrs after the transfection.

### Examination of CSF-1R expression by flow cytometry

Control cells, cells transfected with non-coding and *csf-1r* specific siRNA, and cells treated with 25, 50 or 100 ng/ml CSF-1 (Sigma, USA) were harvested by trypsinization, and incubated for 1 h in 2% FBS (to block unspecific binding sites for antibodies). Then the cells were incubated with 10 μl APC-labeled anti-CSF-1R antibody (eBiosciences, USA) for 1 h at room temperature in the dark. Net, cells were washed with PBS to remove excess antibody and then analyzed using BD FACSCAria II (BD Biosciences, USA) with FACS Diva software (BD Biosciences).

The overlay histograms were created using Flowing Software (Turku University, Finland), http://www.flowingsoftware.com. The experiment was conducted three times.

### Real-time qPCR

Total RNA was isolated using a Total RNA kit (A&A Biotechnology, Poland) according to the manufacturer’s protocol. Isolated RNA samples were dissolved in RNase-free water. The quantity of isolated RNA was measured using NanoDrop (NanoDrop Technologies, USA). The mean concentration of RNA was 140 ng/μl, and A260/280 ratio was between 1.8 and 2.0. The samples with adequate amounts of RNA were treated with DNaseI to eliminate DNA contamination. The samples were subsequently purified using RNeasy MiniElute Cleanup Kit (Qiagen). Finally RNA samples were analyzed on a BioAnalyzer (Agilent, California, USA) to measure final RNA quality and integrity. Only RNA with RIN (RNA Integrity Number) > 9 was used for the further analyses.

Primers used to detect the expression of *csf-1r* gene were designed using PRIMER3 software (free on-line access) and checked using Oligo Calculator (free on-line access) and Primer-Blast (NCBI database). The used sequences were as follow: TGCAGTTTGGGAAGACTCTC and TGTGGACTTCAGCATCTTCA. The optimal annealing time was 4 sec, whereas optimal annealing temperature was 72°C, the detailed description of the optimal time and temperature conditions for the PCR were describe in our previous paper [4]. *rps19* and *hprt* genes were used as non-regulated references for the normalization of target gene expression. Primers sequences and reaction conditions were described in our previously published studies [8-10].

Quantitative RT-PCR was performed using fluorogenic SYBR Green and the Sequence Detection System, Fast 7500 (Applied Biosystems). Data analysis was carried out using the 7500 Fast System SDS Software Version 1.4.0.25 (Applied Biosystems, USA). The results were analyzed using comparative Ct method [15]. Relative transcript abundance of the gene equals ΔCt values (*Δ*Ct = Ct^reference^–Ct^target^). Relative changes in transcript were calculated as ΔΔCt values (*ΔΔ*Ct = *Δ*Ct^normal conditions^–*Δ*Ct^siRNA treated^). The experiment was conducted three times.

### Western blot

To assess activation state of the CSF-1R, the Western blot analyses were conducted. Control cells, cells deprived of FBS (24 hrs) and cells deprived of FBS and supplemented with CSF-1 (24 hrs) were subjected to the expression analysis of p-CSF-1R (Tyr 723) and unfosphorylated form of this protein.

The cells were pelleted by centrifugation at 400 g at 4°C for 5 min. Protein extracts from cultured cells were isolated by lysis of the collected pellets with RIPA buffer (50 mM Tris, pH 7.5, 150 mM NaCl, 1 mM EDTA, 1% NP-40, 0.25% Na-deoxycholate and 1 mM PMSF) supplemented with protease inhibitor cocktail (Sigma-Aldrich) and phosphatase inhibitor cocktail (Sigma-Aldrich) for 30 min at 4°C. Lysates were cleared for 20 min at 14000 rpm, and supernatants were collected. Protein concentration in the lysates was determined by Bio-Rad Protein Assay Dye (Bio-Rad Laboratories Inc., USA). Proteins (50 μg) were resolved by SDS-PAGE and transferred onto PVDF membrane (Sigma-Aldrich). For immunostaining the membranes were blocked with 5% nonfat dry milk in TBS, containing 0.5% Tween 20. The membranes were incubated with primary antibodies: p-CSF-1R (Tyr 723) (Bioss, USA) (1:200) and unphosphorylated CSF-1R (Abcam, UK) (1:200) at 4°C overnight. After the incubation, the membranes were washed 3 times in 1x TBS containing 0.5% Tween 20 and incubated for 1 h at room temperature with secondary antibodies conjugated with appropriate IR fluorophores: IRDye® 800 CW (IR- longer-wavelength near-infrared) (1:5000 dilution). Odyssey Infrared Imaging System (LI-COR Biosciences) was used to analyze the protein expression. Scan resolution of the instrument was set at 169 μm and the intensity at 4. Quantification of the integrated optical density (IOD) was performed with the analysis software provided with the Odyssey scanner (LI-COR Biosciences, USA). To remove antibodies, membranes were incubated 15 min at RT in Restore Western Blot Stripping Buffer (Thermo Scientific, USA). The optical density of band reflecting p-CSF-1R expression was calculated versus band reflecting unphosphorylated CSF-1R expression. This experiment was conducted at least in triplicate.

### Immunohistochemistry

The activation status of CSF-1R was also assessed immunohistochemically. The control cells, cells deprived of FBS (24 hrs) and cells deprived of FBS and supplemented with CSF-1 (24 hrs) were cultured on 4-chambers Lab Tek (Nunc Inc., Denmark) and fixed with 70% ethanol. After washing with TRIS (Dako) the samples were incubated in Peroxidase Blocking Reagent (Dako) for 10 min at room temperature. After 30 min incubation in 5% bovine serum albumin (Sigma Aldrich) the rabbit polyclonal anti- p-CSF-1R (Tyr 723) antibodies (Bioss, USA) diluted in 1% bovine serum were used, and then the slides were incubated at +4°C overnight. For the staining the EnVision kit (Dako) was used (Labelled Polymers consist of secondary anti-rabbit antibodies conjugated with the Horseradish peroxidase-HRP enzyme complex). To develop the colored product the 3,3^′^-Diaminobenzidine (DAB) substrate (Dako) was used. Finally, the haematoxylin was used for nuclei counterstaining.

For immunohistochemical experiment, the negative control samples stained without the use of primary antibodies were set aside. Four independent experiments were conducted.

Ten pictures of each slide were taken using Olympus microscopy BX60. The colorimetric intensity of the IHC-stained antigen (brown precypitate) was counted by a computer-assisted image analyzer (Olympus Microimage™ Image Analysis, software version 4.0 for Windows, USA). The antigen spot color intensity is expressed as mean pixel Integrated Optical Density (IOD).

### Apoptosis assay

The Annexin V-FITC and propidium iodide (PI) dual staining was applied for apoptosis analysis. Control cells and cells treated with: (1) non-coding siRNA and transfection reagent, (2) *csf-1r* specific siRNA, (3) CSF-1, were harvested by trypsinization. These cells, as well as the cells floating in medium (RPMI 1640 containing 10% FBS) were stained using an Annexin V Kit (Becton Dickinson, USA), according to the manufacturer’s protocol. The cells were analyzed by flow cytometer (BD FACS Aria II, Becton Dickinson, USA) within 1 h after staining. Early apoptotic cells with exposed phosphatidylserine but intact cell membranes bound to Annexin V-FITC but excluded PI. Cells in late apoptotic stages were labeled with both Annexin V-FITC and PI, whereas necrotic cells were labeled with PI only. All samples were assayed in triplicate. The experiment was conducted twice.

### Ki-67 expression analysis

The expression of nuclear antigen Ki-67 was measured. Ki-67 is an antigen expressed by cells, which are in the active phases of cell cycle (in each phase except G0). Control cells and cells treated with: (1) non-coding siRNA and transfection reagent, (2) *csf-1r* specific siRNA, (3) CSF-1, were harvested by trypsinization. These cells, as well as the cells floating in medium (RPMI 1640 containing 10% FBS) were fixed in ice-cold ethanol (70%). After fixation, cells were incubated with FITC conjugated anti Ki-67 antibodies (clone B56), or control isotype immunoglobulin IgG1, ĸ (Becton Dickinson). Nuclei were stained with propidium iodide (PI) (Sigma) according to the manufacturer’s protocol. The cells were analyzed using FACS Aria II (Becton Dickinson). All samples were assayed in triplicate. The experiment was conducted twice.

### Wound-healing assay

To assess the role of CSF-1R in migration of cancer cells we applied a wound-healing test. The cancer cells (control cells, transfected and CSF-1 treated) were separately seeded in multi-well plates and cultured in RPMI 1640 containing 10% FBS, until reaching confluence, and then a straight scratch (simulating a wound) was made using a pipette tip (100 ul). The images were captured at the beginning of the test and at regular intervals (after 2, 4 and 6 hours) during cell migration to close the wound. Then the images were compared to quantify migration rate of the cells. This method is particularly suitable to study the cell-cell interactions and cell migration [16]. The pictures were analyzed using a computer-assisted image analyzer (Olympus Microimage™ Image Analysis, software version 4.0 for Windows, USA). All samples were assayed in duplicate. The experiment was conducted three times.

### 3D culture

Cancer control cells and cells treated with: (1) non-coding siRNA and transfection reagent, (2) *csf-1r* specific siRNA, (3) CSF-1 were treated with trypsin and resuspended in culture medium (RPMI 1640 containing 10% FBS). 35 mm culture plates (Corning Inc.) were coated with 100 μl of growth factor reduced Matrigel (BD Biosciences) and left to solidify for 30 min. at 37°C. The cells were then plated at a concentration of 10^4^ cells/ml. The growth of cells on Matrigel was observed everyday under phase-contrast microscope. The experiment was conducted three times.

### Invasion assay

BD BioCoat Matrigel™ invasion chambers (BD Biosciences, USA) pre-coated with BD Matrigel matrix were used according to the manufacturer’s protocol. The assay insert plates were prepared by rehydrating the BD Matrigel Matrix coating with phosphate buffered saline for two hours at 37°C. The rehydration solution was carefully removed, 2.5x10^5^ of control cancer cells, transfected cells and CSF-1 treated cells suspended in RPMI 1640 medium without FBS were added separately onto the apical chambers, and 0.75 ml of RPMI-1640 containing chemoattractant (10% FBS) was added to the basal chambers. Uncoated insert plates, included as invasion controls, were used without rehydration. Assay plates were incubated for 22 hrs at standard culturing conditions. 2.5 μg/ml Calcein AM were added to 20 μl DMSO and then, 10 μl was transferred to 12 ml Hanks Buffered Saline Dispense. 0.5 ml Calcein solution was then transferred into each well of 24-well plate. The medium from insert was removed and multiwell inserts were transferred to the plate containing 0.5 ml/well calcein. Plates were incubated for 1 h at standard culture conditions. The fluorescence of invaded cells was measured with excitation wave length 485 nm and emission wave length Em 530 nm using Tecan Infinite 200 Reader (Tecan). Each condition (cell line and treatment) was assayed three times. The experiment was conducted three times.

### Statistical analysis

The analysis for statistical purposes was conducted using Prism version 5.00 software (GraphPad Software, USA). The one-way ANOVA and Tukey HSD (Honestly Significant Difference) post-hoc test, Dunnett’s test and *t*-test were applied as well as regression analysis. The p-value <0.05 was regarded as significant, whereas, p-value <0.01 and p-value <0.001 as highly significant. The data was expressed as means +/− S.D. The *in vitro* wound healing assay was analyzed using two-way RM ANOVA and Bonferroni post-hoc test.

## Results

### Expression of colony stimulating factor receptor 1 in examined cell lines

**Figure 1:**
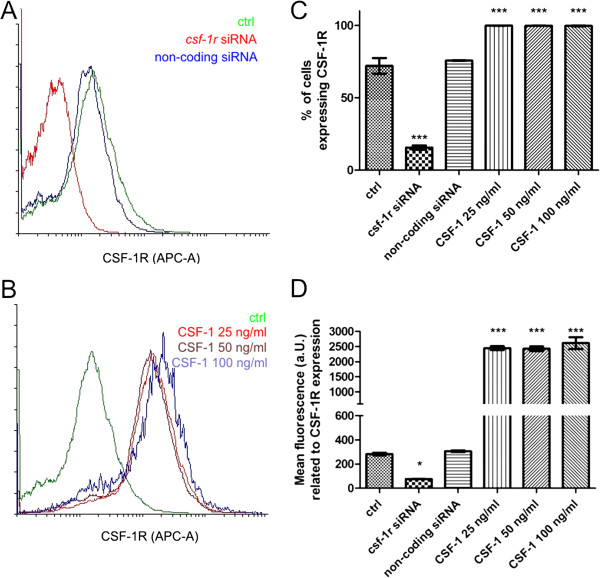
**Expression of CSF-1R at protein level.** Expression of CSF-1R examined in canine mammary cancer cell lines using a BD FACSCAria II (BD Biosciences, USA) and analyzed with FACS Diva software (BD Biosciences). The experiment was conducted three times. **A**. Representative overlay histograms of CSF-1R expression in CMT-U27 canine mammary cancer cells (control), and treated with *csf-1r* specific siRNA and non-coding siRNA. The overlay histograms were created using Flowing Software (Turku University, Finland), www.flowingsoftware.com. **B**. Representative overlay histograms of CSF-1R expression in CMT-U27 canine mammary cancer cells (control), and treated with CSF-1 at the dose of 25, 50 and 100 ng/ml. The overlay histograms were created using Flowing Software (Turku University, Finland), www.flowingsoftware.com. **C**. Representative graphs of the number of CSF-1R expressing cells (CMT-U27) obtained with FACS Aria II (Becton Dickinson). The number of cells which express CSF-1R is significantly decreased after the *csf-1r* specific siRNA treatment and significantly increased after treatment with CSF-1 at the dose of 25, 50 and 100 ng/ml (no significant differences between these doses have been observed). Treatment of cells with non-coding siRNA did not cause any effect. Error bars refer to S.D. p<0.001 was marked as ***. One-way ANOVA followed by Tukey HSD post-hoc test were applied. **D.** Representative graphs of the mean fluorescence related to CSF-1R expression in CMT-U27 cell line obtained with FACS Aria II (Becton Dickinson). The mean expression of CSF-1R is significantly decreased after the *csf-1r* specific siRNA treatment and significantly increased after treatment with CSF-1 at the dose of 25, 50 and 100 ng/ml (no significant differences between these doses have been observed). Treatment of cells with non-coding siRNA did not cause any effect. Error bars refer to S.D. p<0.05 was marked as *, and p<0.001 was marked as ***. One-way ANOVA followed by Tukey HSD post-hoc test were applied.

The expression of CSF-1R at protein level was detected in all examined cell lines (58.7-88.7% of cells expressing CSF-1R), which confirmed our previously published results [8]. We did not observe any correlation between cell line characteristics and the level of CSF-1R expression. Flow cytometric analysis demonstrated that treatment of cells with *csf-1r* specific siRNA significantly decreased the number of cells expressing CSF-1R in all lines. The lowest number of cells expressing CSF-1R after gene knockdown was observed in CMT-U309 cell line (3.0%). At the same time, treatment of cells with scrambled (non-specific) siRNA did not cause any changes in CSF-1R expression which remained equivalent to that of non-transfected control cells. Similarly, culture of cancer cells in serum-starved conditions (RPMI-1640 without addition of FBS) did not affect the number of cells expressing CSF-1R. Analysis using flow cytometry revealed that supplementation of cell cultures with CSF-1 (25 ng/ml, 50 ng/ml or 100 ng/ml) significantly increased the number of cells expressing CSF-1R (Table 1, Figure 1). However, this effect was not dose-dependent, as all used doses caused similar effect (more than 99% of cells expressing CSF-1R).

**Table 1:** Number of cells (%) expressing CSF-1R

	**ctrl**	**Without FBS**	**csf-1r siRNA**	**Non-coding siRNA**	**CSF-1 25 ng/ml**	**CSF-1 50 ng/ml**	**CSF-1 100 ng/ml**
CMT-U27	72.10	73.4	15.65***	75.80	99.85***	99.80***	99.65***
CMT-U309	66.80	70.0	3.00***	64.40	99.75*	99.85*	99.65*
P114	88.70	73.7	38,45***	90.85	99.80*	99.80*	99.90*
CMT-W1	80.45	73.6	23.45***	82.30	99.55**	99.55**	99.75**
CMT-W2	58.70	52.3	23.15***	64.80	99.35***	99.10***	99.55***

The number of CSF-1R expressing cells obtained with FACS Aria II (Becton Dickinson). The number of cells which express CSF-1R is significantly decreased after the *csf-1r* specific siRNA treatment and significantly increased after treatment with CSF-1 at the dose of 25, 50 and 100 ng/ml (no significant differences between these doses have been observed). Treatment of cells with non-coding siRNA did not cause any effect. The experiment has been conducted in three replicates. P<0.05 was regarded as significant and marked as *, whereas p<0.01 and p<0.001 was regarded as highly significant and marked as * and ***, respectively. One-way ANOVA followed by Tukey HSD post-hoc test were applied.

Furthermore, analysis of mean fluorescence related to CSF-1R expression revealed that its expression in cells treated with *csf-1r* specific siRNA decreased 1.5-9.44 fold (Table 2, Figure 1), whereas, in cells treated with CSF-1 the expression increased 5.55-20.05 fold. No significant differences in the number of cells expressing CSF-1R, and its mean expression were noted between the cells exposed to different CSF-1 doses, therefore, in all further experiments the lowest dose was used (25 ng/ml). Cancer cells culture in serum-starved conditions (RPMI-1640 without addition of FBS) decreased the mean expression of CSF-1R 1.17-2.29 fold (Table 2).

**Table 2:** Mean fluorescence related to CSF-1R expression

	**ctrl**	**Without FBS**	***csf-1r *siRNA**	**Non-coding siRNA**	**CSF-1 25 ng/ml**	**CSF-1 50 ng/ml**	**CSF-1 100 ng/ml**
CMT-U27	284.0	191.0*	76.00**	306.5	2450***	2430***	2616***
CMT-U309	255.0	217.5*	107.0***	251.0	5114***	4080***	4100***
P114	458.5	200.0*	48.50**	537.0	4680***	4638***	6178***
CMT-W1	335.0	190.0*	218.5*	383.5	1861**	2268**	1780**
CMT-W2	195.5	130.8*	80.50*	228.5	2448***	2200***	2679***

Mean fluorescence related to CSF-1R expression obtained with FACS Aria II (Becton Dickinson). Mean fluorescence of CSF-1R is significantly decreased after the *csf-1r* specific siRNA treatment and significantly increased after treatment with CSF-1 at the dose of 25, 50 and 100 ng/ml (no significant differences between these doses have been observed). Treatment of cells with non-coding siRNA did not cause any effect. The experiment has been conducted in three replicates. P<0.05 was regarded as significant and marked as *, whereas p<0.01 and p<0.001 was regarded as highly significant and marked as * and ***, respectively. One-way ANOVA followed by Tukey HSD post-hoc test were applied.

**Figure 2:**
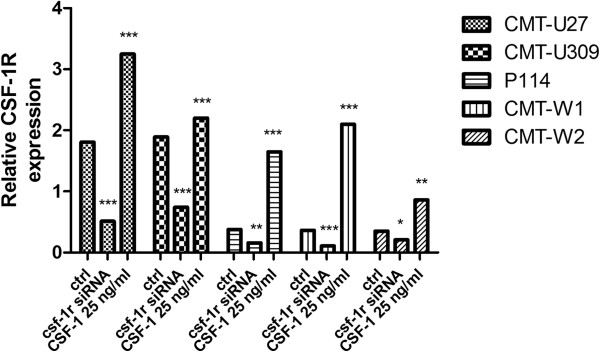
**Expression of CSF-1R at mRNA level.** The relative gene expression level *csf-1r* in CMT-U27, CMT-U309, P114, CMT-W1 and CMT-W2 cell lines in control conditions, after siRNA transfection (to knockdown *csf-1r* expression) and CSF-1 treatment (25 ng/ml) determined using Real-time PCR. The results are presented as means from 3 experiments. Error bars refer to S.D. The statistical analysis was performed using Prism version 5.00 software (GraphPad Software, USA). The one-way ANOVA and Tukey HSD post-hoc test were applied. p<0.05 was regarded as significant and marked as *, whereas p<0.01 and p<0.001 was regarded as highly significant and marked as ** and ***, respectively.

Real-time PCR analysis confirmed a significant decrease in the level of *csf-1r* mRNA in all cell lines treated with *csf-1r -*specific siRNA, whereas the expression of this receptor was significantly increased in all the cell lines treated with CSF-1 (Figure 2).

**Figure 3:**
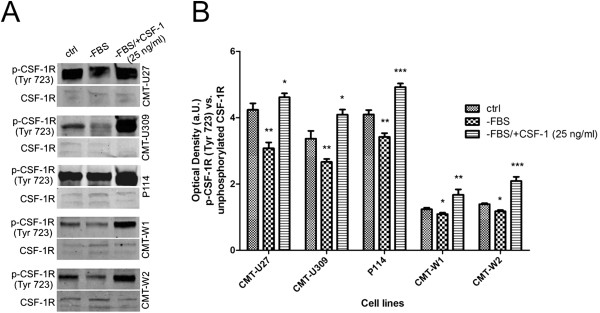
**Examination of CSF-1R activation by Western blot.** Western blot pictures (**A.**) and graph (**B.**) of the CSF-1R activation status in canine mammary carcinoma cell lines in control conditions (ctrl), in serum-starving conditions (−FBS) and in serum-starving conditions supplemented with CSF-1 (25 ng/ml) (−FBS/+CSF-1 25 ng/ml). The level of examined proteins was expressed as IOD (integrated optical density) of protein in arbitrary units with the value obtained using the Odyssey Infrared Imaging System (LI-COR Inc., USA). The results are expressed as the mean ±SD. The Tukey post-hoc test + ANOVA were applied (Graph Pad v. 5.0), the values differed significantly (p≤0.05) were marked as *, whereas values differed highly significant (p≤0.01 and p≤0.001) were marked as ** or ***, respectively.

**Figure 4:**
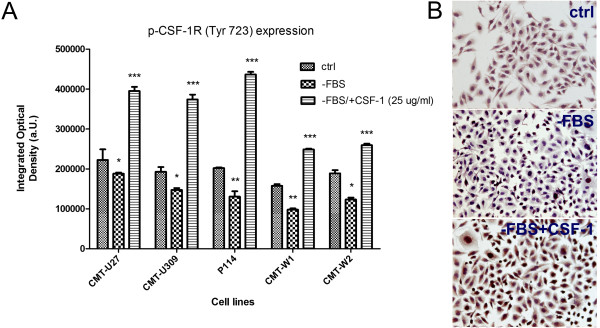
**Examination of CSF-1R activation by Immunohistochemistry. A**. The graph of mean optical density of p-CSF-1R (Tyr 723) in canine mammary cell lines (CMT-U27, CMT-U309, P114, CMT-W1 and CMT-W2) grown in control conditions (ctrl), serum-starving conditions (−FBS) and in serum-starving conditions and supplemented with CSF-1 (25 ng/ml) (−FBS/+CSF-1 25 ng/ml). **B**. Representative pictures of p-CSF-1R expression in CMT-W1 cell line (in control conditions, serum-starving conditions and serum starving conditions and supplemented with CSF-1 (25 ng/ml) obtained using Olympus BX60 microscope (at the magnification of 200x). The p-CSF-1R is reflected as brown precipitate. Ten pictures in each slide were analyzed. The colorimetric intensity of the IHC-stained antigen spots was counted by a computer-assisted image analyzer (Olympus Microimage™ Image Analysis, software version 4.0 for Windows, USA) and the antigen spot color intensity is expressed as mean pixel integrated optical density (IOD). The statistical analysis was performed using Prism version 5.00 software (GraphPad Software, USA). The ANOVA + Tukey post-hoc tests were applied to analyze the optical density in cell lines. p<0.05 was regarded as significant and marked as *, whereas values differed highly significant (p≤0.01 and p≤0.001) were marked as ** or ***, respectively.

Western blot and immunohistochemical analyses of CSF-1R activation showed that in cells deprived of serum, phosphorylation of CSF-1R (Tyr 723) significantly decreased (Figures 3 and 4), whereas in cells deprived of serum but supplemented in CSF-1 (25 ng/ml) CSF-1R activation significantly increased (Figures 3 and 4). The highest level of CSF-1R activation upon CSF-1 treatment was observed in CMT-U309, CMT-U309 and P114 cell lines (Figures 3 and 4).

### CSF-1R inhibits apoptosis and promotes proliferation of canine mammary cancer cells

**Figure 5:**
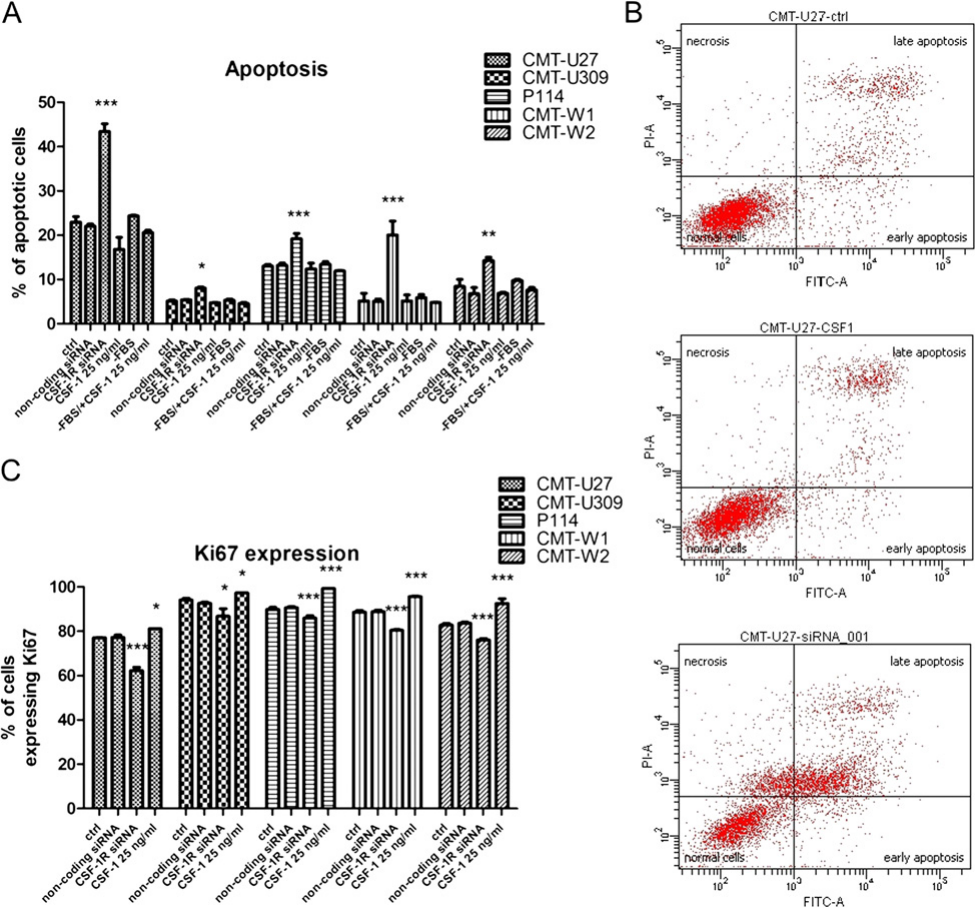
**CSF-1R inhibits apoptosis and promotes proliferation in canine mammary cancer cells.** The effect of *csf-1r* specific siRNA and CSF-1 treatment of canine mammary cancer cell lines (CMT-U27, CMT-U309, P114, CMT-W1 and CMT-W2) on apoptosis (**A**). The number of apoptotic cells represented as a percentages of Annexin-V-positive cells obtained with FACS Aria II (Becton Dickinson) is significantly increased after *csf-1r* silencing. Treatment of cells neither with non-coding siRNA with transfection reagent nor with CSF-1 cause any significant effect comparing to the control. Similarly, cell culture neither in serum-starved conditions (RPMI-1640 without FBS) nor in serum-starved conditions with CSF-1 caused any effect. Error bars refer to S.D. p<0.05 was marked as *, p<0.01 was marked as ** and p<0.001 was marked as ***. One-way ANOVA followed by Tukey HSD post-hoc test were applied. (**B**) The representative histograms and cytograms of CMT-U27 cell line double stained with AnnexinV-FITC and PI. Cells located on the right side of the histograms represented apoptotic cells. On the cytograms are showed normal, early apoptotic, late apoptotic and necrotic cells. Left bottom quadrant shows normal cells, top left quadrant shows necrotic cells (damaged cell membrane but no phosphatydilserine exposure), right bottom quadrant shows the early apoptotic cells (intact cell membrane) and top right quadrant shows cells in late stage of apoptosis. The effect of *csf-1r* gene silencing and CSF-1 (25 ng/ml) treatment on proliferation (**C**) of canine mammary cancer cells was assessed by Ki67 test (BD Bioscience, USA). The number of cycling cells represented as a percentage of Ki67-positive cells is significantly decreases after the transfection whereas significantly increased after CSF-1 treatment. Error bars refer to S.D. The statistical analysis was performed using Prism version 5.00 software (GraphPad Software, USA). One-way ANOVA followed by Tukey HSD post-hoc test were applied. p<0.05 was marked as *, p<0.01 was marked as ** and p<0.001 was marked as ***.

Annexin V analysis revealed that *csf-1r* specific siRNA treatment increased the number of apoptotic cells in all investigated cell lines (Figure 3A, B). The apoptotic effect was highly significant (p<0.001) in CMT-U27 and CMT-W1 cell lines (20.5% and 14.85% increase in number of apoptotic cells, respectively). In CMT-U309, P114 and CMT-W2 cell lines the increase in the number of apoptotic cells was less pronounced, however, also significant (2.9%, 6.2% and 5.8%, respectively). This effect was not obtained when the cancer cells were treated with scrambled siRNA. In this case, the obtained results were comparable to the non-transfected controls. Similarly, treatment of cells with CSF-1 did not cause any significant effect, however in CMT-U27, CMT-U309, P114 and CMT-W2 a decrease in the number of apoptotic cells was noted. Cells cultured in serum-starved conditions with/without CSF-1 (25 ng/ml) also did not show any significant difference in the rate of apoptosis in comparison to control conditions (Figure 5).

Treatment of cells with *csf-1r-*specific siRNA significantly decreased their proliferation, assessed based on the number of Ki-67-positive cells detected using flow cytometric analysis. The highest effect was observed in CMT-U27 (14.8% decrease in the number of Ki67-expressing cells). In case of CMT-U309, P114, CMT-W1 and CMT-W2 cell lines the decrease amounted to: 7.35%, 3.95%, 8.15% and 6.6%, respectively (Figure 5C).

The stimulating effect of CSF-1 on cell proliferation was also observed. The highest increase in number of Ki67-positive cells was observed in CMT-W2, P114 and CMT-W1 cell lines (9.75%, 9.4% and 7%, respectively). In CMT-U27 and CMT-U309 cell lines the increase was less pronounced, namely: 4% and 3.15% respectively (Figure 5C).

### CSF-1R enhances canine mammary cancer cells migration

**Figure 6:**
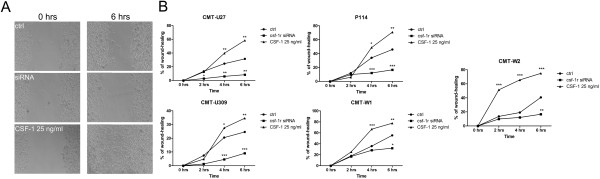
**Role of CSF-1R signaling in canine mammary cancer cells migration assessed by “wound healing” assay. A**. Representative pictures of migration (wound closing) of CMT-U27 cell line: control cells, transfected cells (with *csf-1r* specific siRNA) and cell treated with CSF-1 (25 ng/ml) at 0 and 6 hrs after the scratch was made. **B**. The graphs of % of wound closure after the 2, 4 and 6 hrs of migration. The pictures were taken using phase-contrast microscopy (Olympus). The statistical analysis was performed using Prism version 5.00 software (GraphPad Software, USA). The one-way ANOVA was applied to analyze the results. p<0.05 was regarded as significant and marked as *, whereas p<0.01 and p<0.001 were regarded as highly significant and marked as ** and ***, respectively.

The wound healing assay showed that in all examined cancer cell lines the knockdown of *csf-1r* decreased migratory abilities, whereas treatment with CSF-1 (25 ng/ml) increased migration (Figure 6). CMT-U27 cells grown in the presence of CSF-1 closed 58.4% of the wound after six hours, whereas the control CMT-U27 cells closed only 31.49% of the wound during the same period. Furthermore, treatment of CMT-U27 cells with *csf-1r-*specific siRNA resulted in a significant decrease in wound healing abilities of these cells, as only 8.42% of the wound was closed after six hrs. In case of P114, CMT-W1 and CMT-W2 cells cultured with CSF-1 a significant percent of wound was closed after 6 hrs (70.88, 77.54 and 86.79, respectively), whereas the control cells closed only 45.89%, 55.4% and 74.91% of the wound, respectively. When these cell lines were treated with *csf-1r* siRNA not more than 16.62%, 31.95% and 40.63% of the wound was closed after six hrs, respectively. CMT-U309 cells treated with CSF-1 closed 34.62% of the wound after six hrs, whereas the control cells closed only 24.62% and *csf-1r* siRNA treated cells showed only 9.06% of wound closure.

### CSF-1R increases canine mammary cancer cells invasion in matrigel matrix

**Figure 7:**
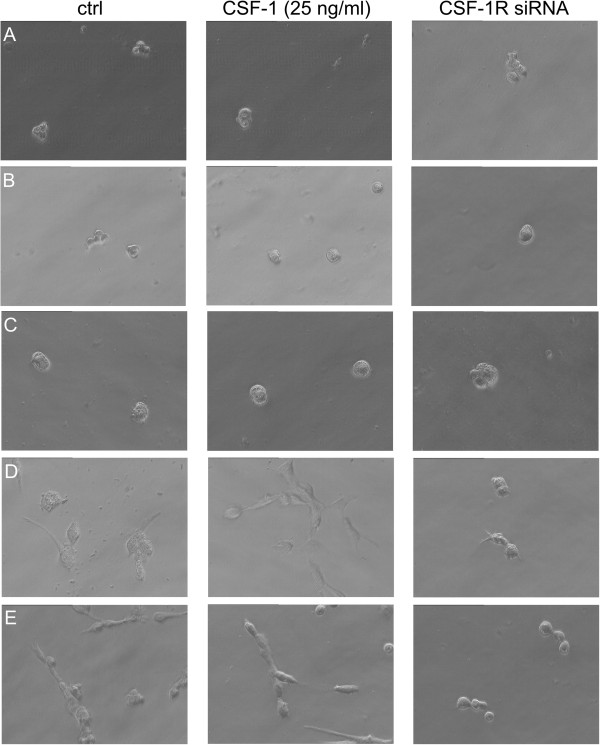
**An impact of csf-1r silencing and CSF-1 treatment on 3D growth of canine mammary cancer cell lines.** Growth characteristics of CMT-U27 (**A**), CMT-U309 (**B**), P114 (**C**), CMT-W1 (**D**) and CMT-W2 (**E**) cell lines (phase contrast micrographs) grown on Matrigel matrix for 24 hours in control conditions, transfected with csf-1r specific siRNA and treated with CSF-1 (25 ng/ml).

We have assessed the impact of *csf-1r* knockdown and CSF-1 supplementation on growth characteristics of canine mammary cancer cells on Matrigel matrix (Figure 7). After 24 hrs of culturing on Matrigel CMT-U27, CMT-U309 and P114 cell lines formed colonies, whereas CMT-W1 and CMT-W2 cell lines formed branching structures, which could result from their invasive phenotype, described elsewhere [8]. However, we observed that *csf-1r* silencing decreased the ability of CMT-W1 and CMT-W2 cells ability to invade the Matrigel and changed their growth characteristics (they formed colonies not the branching structures). The addition of CSF-1 to the culture did not cause any effect on the growth characteristics of the examined cell lines.

**Figure 8:**
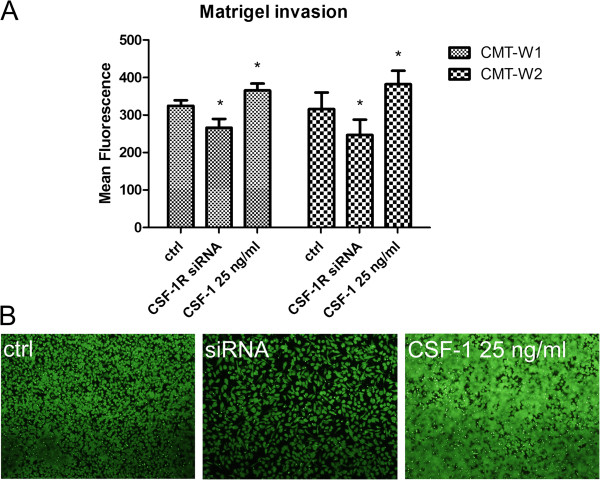
**Invasion assay of highly invasive canine mammary cancer cell lines after treatment with *csf-1r *specific siRNA and CSF-1 (25 ng/ml). A**. The graph of fluorescence intensity related to invasion of canine mammary cancer cell lines (CMT-W1 and CMT-W2) grown in control conditions (ctrl), transfected with *csf-1r* specific siRNA and treated with CSF-1 (25 ng/ml). The statistical analysis was performed using Prism version 5.00 software (GraphPad Software, USA). Error bars refer to S.D. The unpaired t-test was applied to analyze the results. p<0.05 was regarded as significant and marked as *. **B**. Pictures of CMT-W1 cells (green fluorescence) obtained using Olympus BX60 microscope examined in control conditions and after transfection (*csf-1r* specific siRNA) and CSF-1 (25 ng/ml) treatment.

Invasion assay of the most invasive cell lines CMT-W1 and CMT-W2 showed that CSF-1 increased, whereas *csf-1r* knockdown decreased their ability to invade the Matrigel (Figure 8). The fluorescence intensity related to the migration of CMT-W1 control cells was 324.8, whereas in case of the CMT-W1 cells treated with *csf-1r* siRNA and CSF-1 (25 ng/ml) it was 266.07 and 365.53, respectively. The fluorescence intensity related to the migration of CMT-W2 control cells was 316. When these cells were transfected with *csf-1r-*specific siRNA, the intensity decreased to 247.07, whereas the treatment with CSF-1 resulted in an increase of the fluorescence intensity to 382.14.

## Discussion

Our results showed that all the examined canine mammary cancer cell lines expressed CSF-1R. This finding is in accordance with previously published results, which showed that CSF-1R is expressed in 100% of canine mammary cancers. Moreover, its expression increased in more malignant and metastatic tumors [10]. However, in humans CSF-1R is expressed in only about 50% of breast cancers [17]. It suggests that CSF-1R can be even more important for canine mammary cancer development than for breast cancer. We suppose that the differences between CSF-1R expression in dogs and humans may results from the specificity of canine ovarian cycle, as there are significant differences in the course of this cycle among mammalian species. In a non-pregnant bitch, estrus is usually followed by a prolonged luteal phase correlating with pregnancy-like concentrations of hormones [14]. This prolonged exposure to serum progesterone stimulates mammary gland development, which is very often accompanied with subsequent lactation [18]. This pseudo pregnancy is often followed by development of mammary tumors [19]. Because CSF-1R is expressed in mammary tissue during pregnancy and lactation [6], canine epithelial cells may show its expression more often than in humans.

However, the role of CSF-1R in canine mammary tissue or cancer has not been investigated yet. Thus, the aim of our study was to evaluate the role of CSF-1R in biology of canine mammary cancer cells. We used five various cell lines and treated them with 1) *csf-1r* specific siRNA (to knockdown its expression) and with 2) CSF-1 to stimulate the expression of this receptor. Cells were grown in complete medium for 24 hrs. Then, they were incubated in the absence of FBS for 24 hrs before being incubated in RPMI 1640 with 10% FBS and CSF-1 at the concentration of 25 ng/ml, 50 ng/ml and 100 ng/ml for further 24 hrs (these doses have been described by other authors, as the most effective ones [6,20]). Flow cytometric analyses revealed that the siRNA sequences used in this study decreased both: the number of cells expressing CSF-1R and mean fluorescence related to its expression (Tables 1 and 2; Figure 1). We also observed that serum-starved conditions (RPMI-1640 without addition of FBS) caused a significant decrease in the mean expression and phosphorylation of CSF-1R in cancer cells (Figures 3 and 4), whereas it did not affect the number of cells expressing CSF-1R. These results suggest that FBS can contain CSF-1 or other growth factors that stimulate CSF-1R. However, the autocrine stimulation of cancer cells cannot be ruled out. Supplementation of cells growth medium or medium without FBS with CSF-1 caused an significant increased CSF-1R expression and phosphorylation (Tables 1 and 2; Figures 1, 2, 3 and 4), however not in a dose dependent manner. That is why we decided to use the lowest effective dose: 25 ng/ml, in following experiments. Similarly, this dose was used by other authors for assessment of the role of CSF-1R in biology of breast cancer cell lines [6].

The impact of RNA interference and CSF-1 supplementation on *csf-1r* expression was also confirmed using Real-time qPCR. However, there were some differences between the observed level of *csf-1r* mRNA and CSF-1R protein. These results are not surprising, as it has been reported that in experiments with the use of RNAi the correlation between the gene and protein expression levels can be poor, although the compatibility between these two levels of expressions may be good, based on ‘present’ *vs*. ‘absent’ findings [21,22].

We found that both: knockdown of *csf-1r* and an increase of its expression after CSF-1 treatment have a significant impact on cancer cells. The treatment of cell with CSF-1 mimicked the presence of macrophages in the tumor microenvironment, as our previous findings have demonstrated that the presence of macrophages in the tumor microenvironment increases CSF-1R expression in cancer cells [8].

The results of our present study showed that knockdown of CSF-1R increased the number of apoptotic cells (Figure 5A, B). This was not related with the toxic effect of transfection reagents or procedure because in cells treated with non-coding siRNA and transfection reagent the number of apoptotic cells did not differ from the control cells. CSF-1 treatment neither caused any significant effect on apoptosis in cancer cells grown in normal medium, nor in cells grown in serum-starved conditions. Interestingly, blockade of CSF-1/CSF-1R signaling increased the number of apoptotic cells dramatically. Up-to-date there are no available reports regarding the impact of CSF-1R on apoptosis in cancer cells. However, an interesting study showed that blockade of CSF-1R by BAY 43-9006/Sorafenib increased apoptosis in Hodgkin-/Reed-Sternberg cells [23] and it enhanced the activity of conventional chemotherapeutics.

In all examined cell lines, we observed an increase in proliferation induced by CSF-1. The number of cells expressing Ki67 antigen was almost 10% higher in P114 and CMT-W2 cell lines (Figure 5C). However, CSF-1R silencing significantly decreased proliferation in all examined cell lines. The number of cells expressing Ki67 antigen was 15% decreased in CMT-U27 cell line, whereas in the rest of cell lines the decrease was of 4-8% (Figure 3C). It has been shown previously that in breast cancer cell lines administration of CSF-1 activates cyclin D1, as a consequence of ERK1/2 activation [17,24].

There are few reports of CSF-1R role in tumor metastasis. Sapi et al. [25] injected CSF-1R overexpressing cancer cells into mice and observed increased lung metastasis in these animals. Our own results also confirm these findings, as we observed the highest expression of CSF-1R in the most malignant and metastatic mammary tumors [10]. Results of prof. Condeelis group [9] indicate that both autocrine and paracrine CSF-1 loops significantly contribute to dissemination of cancer cells and progression in human breast cancer.

Considering invasion and migration as steps in the metastatic cascade our results confirm that CSF-1R signaling promotes this process. We showed that treatment of cells with CSF-1 as well as *csf-1r* knockdown had significant effect on cancer cells migration. The “wound healing” assay showed that *csf-1r* specific siRNA treatment of cells significantly inhibited cancer cells migration (about 15-58% difference in “wound” closure between control cells and transfected cells) (Figure 6). On the other hand, CSF-1 treatment increased cells migration, causing about 10-34% difference in “wound” closure between control cells and cells treated with CSF-1 (Figure 6). These findings are compatible with the results of our previous study on the effect of co-culture of cancer cells and tumor-associated macrophages. We showed that the presence of macrophages in cancer cell microenvironment accelerated “wound” closure of about 30% [8]. Based on these findings we can conclude that the used CSF-1 dose mimicked the presence of macrophages in cancer cells environment regarding the CSF-1R signaling.

Our results showed also that *csf-1r* RNAi knockdown caused significant changes in growth characteristics on Matrigel matrix of canine cancer cells (Figure 7). The growth characteristics of examined cells on extracellular matrix components have previously been published [4]. The present results showed that only two of the cell lines form branches and invade matrix. These two highly invasive cell lines have been previously described as “metastatic” [8]. Knockdown of *csf-1r* caused changes in growth of these two highly invasive cell lines, as most of them did not invade Matrigel matrix or form branches, but grew in colonies. The cells were seeded on Matrigel matrix 48 hrs after transfection and then observed after the next 24 hrs (that is: 72 hrs post transfection). As siRNA gene silencing is a transient effect [23], after the next 24 hrs they started to grow in their normal manner (data not showed). Treatment of cells with CSF-1 did not cause any visible effect on examined cell lines (Figure 7). This is a good method to assess if the cell line invades the matrix or not, however, this is not an objective method to assess the level of invasion. To assess more accurately the effect of CSF-1 on canine mammary cancer cells invasion, we conducted the invasion assay of highly invasive cell lines in Boyden chambers (Figure 8). Our results confirmed that the decreased CSF-1R expression is accompanied by the lower ability of cancer cells to invade matrix, whereas the presence of CSF-1 in the culture medium increased canine mammary cancer cells invasion in Matrigel matrix (Figure 8).

The findings of our study suggest that blocking of CSF-1R in patients may suppress tumor development and invasion by acting on cancer cells itself. Moreover, because mammary tumor microenvironment is rich in tumor-associated macrophages, which express CSF-1R (they have resident-type macrophage phenotype [26]), this approach could also affect tumor-associated macrophages that are an important element in cancer development and invasion.

## Conclusions

In conclusion, the evidence of the expression and functional role of the CSF-1R in canine mammary cancer cells indicate that CSF-1R targeting may be a good therapeutic approach.

## Competing interests

The authors declare that they have no competing interests.

## Authors’ contributions

MK research design, experimental design, FACS analyses, siRNA transfections, real-time qPCR, Western blot analysis, “wound healing” assay, statistical analysis, manuscript preparation, real-time qPCR analysis, KM invasion assay, apoptosis assay, proliferation assay, immunohistochemistry, JM CSF-1R staining, protein isolation, AH and MB Western blot analysis, AK migration assay, invasion assay, MK RNA isolation, examination of CSF-1R expression, KP real-time qPCR analysis, TM manuscript preparation. All authors read and approved the final manuscript.

## References

[B1] Richardsen E Sorbye SW Crowe JP Yang JL Busund LT Expression of M-CSF and CSF-1R is correlated with histological grade in soft tissue tumors Anticancer Res 2009 29 3861 3866 19846920

[B2] Lin EY Pollard JW Role of infiltrated leucocytes in tumor growth and spread Br J Cancer 2004 90 20-53-2058 15164120 10.1038/sj.bjc.6601705 PMC2410285

[B3] Coussens LM Werb Z Inflammation and cancer Nature 2002 420 860 867 10.1038/nature01322 12490959 PMC2803035

[B4] Elgert KD Alleva DG Mullins DW Tumor-induced immune dysfunction: the macrophage connection J Leukocyte Biol 1998 64 275 288 9738653 10.1002/jlb.64.3.275

[B5] Priceman SJ Sung JL Shaposhnik Z Burton JB Torres-Collado AX Moughon DL Johnson M Lusis AJ Cohen DA Iruela-Aripse ML Wu L Targeting distinct tumor-infiltrating myeloid cells by inhibiting CSF-1 receptor: combating tumor evasion of antiangiogenic therapy Blood 2010 115 1461 1471 10.1182/blood-2009-08-237412 20008303 PMC2826767

[B6] Morandi A Barbetti V Riverso M Sbarba PD Rovida E The colony-stimulating factor-1 (CSF-1) receptor sustains ERK1/2 activation and proliferation in Brest cancer cell lines PLoS One 2011 6 1 e27450 10.1371/journal.pone.0027450 22096574 PMC3212567

[B7] Sapi E The role of CSF-1 in normal physiology of mammary gland and breast cancer: an update Exp Biol Med 2004 229 1 11 10.1177/153537020422900101 14709771

[B8] Król M Pawłowski KM Majchrzak K Gajewska M Majewska A Motyl T Global gene expression profiles of canine macrophages and canine mammary cancer cells grown as a co-culture *in vitro* BMC Vet Res 2012 8 16 10.1186/1746-6148-8-16 22353646 PMC3315417

[B9] Patsialou A Wyckoff J Wang Y Goswami S Stanley ER Condeelis JS Invasion of human breast cancer cells in vivo requires both paracrine and autocrine loops involving the colony stimulating factor-1 receptor Cancer Res 2009 69 9498 9506 10.1158/0008-5472.CAN-09-1868 19934330 PMC2794986

[B10] Król M Pawłowski KM Majchrzak K Dolka I Abramowicz A Szyszko K Motyl T Density of tumor-associated macrophages (TAMs) and expression of their growth factor receptor MCSF-R and CD14 in canine mammary adenocarcinomas of various grade of malignancy and metastasis Pol J Vet Sci 2011 14 3 10 21528705 10.2478/v10181-011-0001-3

[B11] Król M Pawłowski KM Skierski J Rao NAS Hellmen E Mol JA Motyl T Transcriptomic profile of two canine mammary cancer cell lines with different proliferative and anti-apoptotic potential J Physiol Pharmacol 2009 60 95 106 19609018

[B12] Król M Pawłowski KM Skierski J Turowski P Majewska A Polańska J Ugorski M Morty RE Motyl T Transcriptomic “portraits” of canine mammary cancer cell lines with various phenotype J Appl Genet 2010 51 169 183 10.1007/BF03195725 20453304

[B13] Król M Polańska J Pawłowski KM Skierski J Majewska A Ugorski M Motyl T Molecular signature of cell lines isolated from mammary adenocarcinoma metastases to lungs J Appl Genet 2010 51 37 50 10.1007/BF03195709 20145299

[B14] Pawłowski KM Popielarz D Szyszko K Motyl T Król M Growth Hormone Receptor RNA interference decreases proliferation and enhances apoptosis in canine mammary carcinoma cell line CMT-U27 Vet Comp Oncol 2012 10 1 2 15 10.1111/j.1476-5829.2011.00269.x 22235976

[B15] Schmittgen TD Livac KJ Analyzing real-time PCR data by the comparative Ct method Nat Prot 2008 3 1101 1108 10.1038/nprot.2008.73 18546601

[B16] Jeffery PL Murray RE Yeh AH McNamara JF Duncan RP Francis GD Herington AC Chopin LK Expression and function of the ghrelin axis, including a novel preproghrelin isoform, in human breast cancer tissues and cell lines Endocr Relat Cancer 2005 12 839 850 10.1677/erc.1.00984 16322325

[B17] Lee AW Nambirajan S Moffat JG CSF-1 activates MAPK-dependent and p53-independent pathways to induce growth arrest of hormone-dependent human breast cancer cells Oncogene 1999 18 7477 7494 10.1038/sj.onc.1203123 10602507

[B18] Sokolowski JH False pregnancy Vet Clin North Am 1982 12 93 98 10.1016/s0195-5616(82)50009-5 6980516

[B19] Gobello C Concannon PW Verstegen J Concannon PW, England G, Verstegen J Canine pseudopregnancy: A Review Recent advances in Small animal reproduction 2011 Ithaca, New York USA Retrieved: International Veterinary Information Services (www.ivis.org)

[B20] Wittrant Y Gorin Y Mohan S Wagner B Abboud-Werner SL Colony-stimulating factor-1 (CSF-1) directly inhibits receptor activator of nuclear factor-kB ligand (RANKL) expression by osteoblasts Endocrinology 2009 150 4977 4988 10.1210/en.2009-0248 19819976 PMC2775986

[B21] Chen G Gharib TG Huang CC Taylor JMG Misek DE Kardia SLR Giordano TJ Iannettoni MD Orringer MB Hanash SM Beer DG Discordant protein and mRNA expression in lung adenocarcinomas Mol and Cel Proteom 2002 1.4 304 313 10.1074/mcp.m200008-mcp200 12096112

[B22] Pascal LE True LD Campbell DS Deutsch EW Risk M Coleman IM Eichner LJ Nelson PS Liu AY Correlation of mRNA and protein levels: cell type-specific gene expression of cluster designation antigens in the prostate BMC Genomics 2008 9 246 10.1186/1471-2164-9-246 18501003 PMC2413246

[B23] Ullrich K Wurster KD Lamprecht B Kochert K Engert A Dorken B Janz M Mathas S BAY 43-9005/Sorafenib blocks CSF1R activity and induces apoptosis in various classical Hodgkin lymphoma cell lines Brit J Haemat 2011 155 395 408 10.1111/j.1365-2141.2011.08682.x 21517818

[B24] Roussel MF Regulation of cell cycle entry and G1 progression by CSF-1 Mol Reprod Dev 1997 46 11 18 10.1002/(SICI)1098-2795(199701)46:1<11::AID-MRD3>3.0.CO;2-U 8981358

[B25] Sapi E Flick MB Rodov S Gilmore-Hebert M Kelley M Rockwell S Kacinski BM Independent regulation of invasion and anchorage-independent growth by different autophosphorylation sites of the macrophage colony-stimulating factor 1 receptor Cancer Res 1996 56 5704 5712 8971179

[B26] Hume DA MacDonald PA Therapeutic applications of macrophage colony-stimulating factor-1 (CSF-1) and antagonists of CSF-1 receptor (CSF-1R) signaling Blood 2011 119 1810 1820 22186992 10.1182/blood-2011-09-379214

